# Radiation-Induced Reactions in the Liver—Modulation of Radiation Effects by Lifestyle-Related Factors—

**DOI:** 10.3390/ijms19123855

**Published:** 2018-12-03

**Authors:** Tetsuo Nakajima, Yasuharu Ninomiya, Mitsuru Nenoi

**Affiliations:** 1 Department of Radiation Effects Research, National Institute of Radiological Sciences, QST, Chiba-shi 263-8555, Japan; ninomiya.yasuharu@qst.go.jp; 2 Human Resources Development Center, National Institute of Radiological Sciences, QST, Chiba-shi 263-8555, Japan; nenoi.mitsuru@qst.go.jp

**Keywords:** radiation, liver damage, inflammation, liver cancer, lifestyle

## Abstract

Radiation has a wide variety of effects on the liver. Fibrosis is a concern in medical fields as one of the acute effects of high-dose irradiation, such as with cancer radiotherapies. Cancer is also an important concern following exposure to radiation. The liver has an active metabolism and reacts to radiations. In addition, effects are modulated by many environmental factors, such as high-calorie foods or alcohol beverages. Adaptations to other environmental conditions could also influence the effects of radiation. Reactions to radiation may not be optimally regulated under conditions modulated by the environment, possibly leading to dysregulation, disease or cancer. Here, we introduce some reactions to ionizing radiation in the liver, as demonstrated primarily in animal experiments. In addition, modulation of radiation-induced effects in the liver due to factors such as obesity, alcohol drinking, or supplements derived from foods are reviewed. Perspectives on medical applications by modulations of radiation effects are also discussed.

## 1. Introduction

The liver is an active metabolic organ that is easily influenced by many environmental conditions. Ionizing radiation is one such environmental factor and living organisms exposed to relatively high-dose radiation can sustain severe damage or die within a short period due to acute effects [1]. Moderately high-dose radiations (such as 2 Gy/day in fractionated irradiation as cancer radiotherapy, etc.) (Gy: gray, absorbed dose) can have different effects [2,3], depending on the exposed tissue and age at exposure [3,4,5]. Concerning the liver, an investigation of survivors from the Hiroshima and Nagasaki atomic bombings found that incidences of cancer and fatty livers were increased [6,7]. In addition, in the case of cancer radiotherapies, although radiation exposure is to a limited area, normal tissues in that area can receive high-dose exposure and the liver could thus sustain acute damage such as fibrosis [3]. Humans encounter various opportunities for exposure to radiation in daily life and these frequently include medical treatments such as radiotherapies and diagnostic modalities. Radiation effects are considered to be dependent on conditions such as species, age and sex. Indeed, in animal experiments, radiation effects are reportedly dependent on strain, age and sex [8,9,10,11,12]. Lifestyle factors are extremely important in human health and are thought to cause around 70% of cancers. To evaluate health effects, lifestyle must be taken into consideration [13].

Radiation effects on the liver may be influenced by lifestyle, particularly obesity, diet and alcohol, each of which are also related to various liver diseases. For example, a survey of Japanese nuclear workers suggested relationships between radiation effects and alcohol drinking [14]. Because the liver is easily influenced by environmental conditions, radiation effects in the liver may be modulated by many possible cases. For example, numerous specific environmental factors are encountered in space (low gravity, specific day-night shift time, radiation, etc.) and extended stays in space are reportedly suggested to increase the risk of non-alcoholic fatty liver disease (NAFLD) in mouse livers [15]. In space, living organisms influenced by environmental factors other than radiation may be exposed to cosmic radiation, including X-rays, gamma rays and particle radiation (proton or heavy-ion charged beams). Analyses of the mechanisms underlying NAFLD may offer a clue to understand radiation effects in the livers.

Here, we review how radiation influences liver function either alone or in combination with lifestyle factors.

## 2. Effects of Radiation on the Liver

In studies using cells or experimental animals, radiation damage such as DNA double-strand breaks and lipid peroxidation have been observed at the molecular levels. Such damage is considered to induce many biochemical reactions, subsequently leading to various disease states. In cancer radiotherapies, liver fibrosis as a form of acute radiation damage has been reported following exposure to high-dose radiation [3]. Moreover, radiation-induced cancers have been observed as late effects [6]. On the other hand, DNA damage repair and anti-oxidative abilities are induced in the liver after irradiation [16,17,18], indicating that the liver has functions to react to radiation, which appear to regulate radio-sensitivity in the liver. Indeed, though parenchymal cells (hepatocytes) in the liver are generally in G0 stage in cell cycle, irradiation induces DNA repair activities via DNA damage even in the non-proliferating cells [19]. The other type cells in the liver also contribute to radiation effects via inflammatory reactions and so forth, after irradiation [20,21]. Interestingly, early period after irradiation, up-regulation of chemokine gene expression was observed only in portal vessel areas but not in the parenchyma [22]. The induced chemokines appear to induce recruitment of inflammatory cells. In addition, ROS induced via hypoxia and so forth, in the vascular endothelium by radiation injury might contribute to chemokine production and subsequent inflammation in the irradiated livers. In experiments using mice, characteristics of radiation effects are dependent on ages, sex and strains [8,9,10,11,12]. In terms of age-dependency, radiation exposure in infancy, as the active, proliferative stage of liver development, appears related to carcinogenesis [11,23]. Moreover, radio-sensitivity leading to liver carcinogenesis appears to differ among mouse strains [8].

The mechanisms of radiation-induced liver cancer have been investigated at the molecular levels. Defects in the *FHIT* gene appear related to an increased frequency of radiation-induced liver and lung cancer [24]. The *FHIT* gene is a tumor suppressor gene that reportedly participates in the regulation of normal cell checkpoint and progression of apoptosis [25]. Many cases in radiation-induced liver cancer are reportedly induced by acute irradiation [9,11,12] but liver carcinogenesis might be also related to radiation-dose rates [10]. In *FHIT*-defective mice, fractionated irradiation does not induce liver cancer. The *FHIT* function may ameliorate damage from acute irradiation but not participate in the repair of damage following low-dose-rate irradiation [24].

Many experiments have examined alterations in mRNA expression after irradiation. How these alterations are related to radiation-induced recovery or damage remains obscure [26,27,28]. On the other hand, much attention has been being given to radiation-induced epigenetic effects like DNA methylation and microRNA regulation, although the contribution remains unclear in the liver. Global DNA methylation has been demonstrated to be lost in the liver as well as other tissues like thymus, spleen or bone marrow following irradiation [29]. However, changes to DNA methylation after irradiation seem also dependent on sex strains and dose/dose rates of radiation [29,30].

As other epigenetic alterations, changes in microRNA (miRNA) expression are interesting and represent a prominent molecular target for medical treatment [31,32]. In the liver, miRNA21 appears related to radiation-induced liver carcinogenesis [33]. In particular, iron ion beam, high-linear energy transfer (LET) particle radiation appears to markedly induce miRNA21 expression. In addition, miRNA34 is reportedly also altered after irradiation [34].

**Figure 1:**
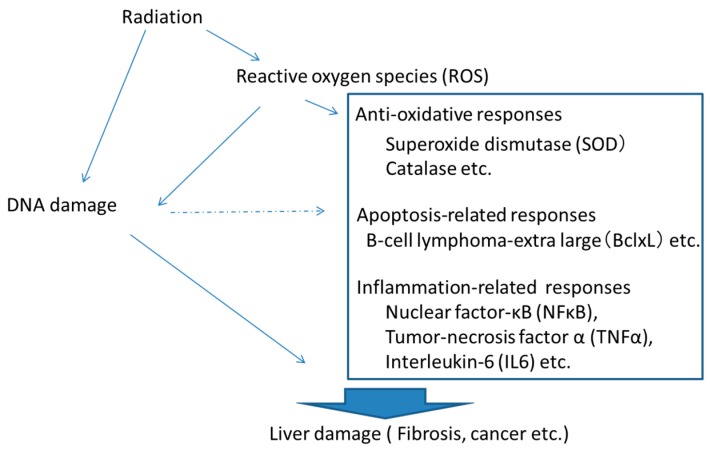
Radiation-induced responses in the liver.

Other studies have examined alterations to protein expression [35,36]. In particular, proteins related to inflammation or apoptosis seem to contribute to the effects of irradiation on the liver [36,37]. High-dose radiation usually induces inflammation, leading to fibrosis or cancer. Interestingly, mice irradiated acutely at high doses (4Gy or 8Gy) showed persistent alterations to expression of proteins related to inflammation, whereas a total dose of 8Gy by low-dose-rate, long-term irradiation induced alteration of expression for many apoptosis-related proteins [36]. Apoptosis was suggested to have a kind of repressive function in liver carcinogenesis [38]. Alteration of apoptosis-related proteins may imply that radiation-induced protective abilities persist following damage from radiation [36]. We have also detected the induction of rhodanese, a participant in sulfur metabolism, following low-dose-rate, long-term irradiation. This enzyme is involved in intracellular redox regulation and may be increased to reduce oxidative stress following irradiation [17]. In addition, the acute phase disulfide-rich plasma glycoprotein, α2-macroglobulin ameliorates acute effects in the liver after irradiation [39]. Taken together, sulfur metabolism may be a critical pathway in reactions to radiation [17]. Moreover, after high-dose irradiation, some cytoprotective reactions including Superoxide dismutase (SOD) activation are induced in livers. Concerning the effects of radiation on nuclear factor-κB ( NFκB )activation, various observations have been reported in early responses to radiation [40,41,42]. Nuclear translocation of NFκB appears to be induced 6–24 h after radiation in mice [41]. In addition, the activation in the expression appears to be induced late time after irradiation in rats [42]. Alteration to many regulators are thus induced to maintain homeostasis or ameliorate damage after irradiation (Figure 1, Table 1).

Epigenetic alterations such as DNA methylation, miRNA regulation and oxidative stress, which are related to inflammation or apoptosis, are undoubtedly involved in radiation damage and subsequent diseases. These alterations and stresses could be usually induced in the metabolizing pathways of the liver in daily life, particularly under conditions of a high-calorie diet or drinking alcohol. Epidemiological data concerning survivors of the Hiroshima and Nagasaki atomic bombings suggest that development in some cancers may be modulated by smoking, drinking alcohol, foods and viral infections, depending on the age at which the survivors were exposed [6]. Diet is also important. Indeed, intakes of vegetables or foods have been suggested to influence cancer incidence among the survivors [44,45].

Radiation is a risk factor of cancer and has also been investigated in terms of relationships with other diseases [46]. In addition, radiation can induce fatty livers [7]. Low-dose-rate irradiation close to the rate of exposure to cosmic radiation on the International Space station (0.5–1 mSv/day) (mSv: millisievert, effective dose) can also induce liver cancer in animal experiments [10]. Interestingly fatty degeneration has been observed before the detection of liver cancer [10,47] A relationship between fatty metabolism alteration and liver cancer has been demonstrated in research into the development of NAFLD, one of lifestyle-related diseases [48]. Acute irradiation induces fat metabolic pathways [49]. Changes in fatty metabolism may be involved in radiation-induced liver carcinogenesis.

Next, in modern life, relationships between radiation effects and the three lifestyle factors of obesity, alcohol intake and intake of food-derived supplements are discussed at the molecular level in liver diseases.

## 3. Radiation Effects and Lifestyle-Related Environmental Stressors

### 3.1. Obesity and High-Calorie Diets

Obesity is considered to be involved in about 20% of cancers [50]. In liver cancer, the higher the body mass index, the greater the cancer risk [48]. Mouse experiments have revealed that a high-fat-diet induces steatosis, inflammation fibrosis, carcinomas [51]. Obesity or a high calorie diet are greater risk factors than radiation but the modulation of radiation effects by those factors remains unclear. A high-calorie diet is known to induce DNA damage in the liver [52] but whether a synergistic effect exists for DNA damage from other mutagens like radiation remains obscure. We have observed that high-fat-diets had no effects on radiation-induced DNA damage in the bone marrow of irradiated mice but clearly modulated radiation effects in the liver at the epigenetic levels [53]. This finding indicates that a high calorie diet or obesity modulate radiation effects in the liver. In addition, DNA repair enzymes such as poly (ADP-ribose) polymerase 1 (PARP1) are modulated by a high-calorie diet and these enzymes are involved in the repair of radiation-induced DNA damage [54,55]. Indeed, DNA damage is related to not only carcinogenesis but also non-cancer diseases. Epidemiologically, investigations of survivors from the Hiroshima and Nagasaki atomic bombings, have suggested that fatty liver and cardiac diseases may be induced by radiation [7,56]. These pathologies are also known to be induced by a high-calorie diet or obesity [57,58]. Concerning metabolic syndrome, atherosclerosis-prone apolipoprotein E (ApoE)(−/−) ataxia telangiectasia mutated (ATM)(+/−) mice were used to investigate the effects of a high-calorie diet on DNA damage and metabolic syndrome in the liver. ATM haplotype(+/−) mice, which show a partial defect in ATM, have defective DNA repair abilities. In the case of ApoE(−/−) ATM(+/−) mice, atherosclerosis was accelerated by a high-calorie diet [59]. In these mice, oxidative stress was increased and DNA damage in mitochondria was induced, resulting in the alteration of metabolic features such as hepatic steatosis and serum liver enzyme activities. Atherosclerosis was also reported to be induced in ApoE(−/−) mice by radiation [60]. These results suggest that high-calorie diets modulate radiation effects such as metabolic diseases through oxidative stress and DNA damage. We have demonstrated that a high-fat diet modulates radio-sensitivity using mouse liver cells. An increased caloric intake was suggested to promote oxidative stress, influencing radio-sensitivities [53]. In addition, low-dose-rate, long-term irradiation induces fatty degeneration before the incidence of liver cancers [47]. The relationship between the metabolic alteration of lipids and liver carcinogenesis has been investigated in detail for NAFLD [48]. Overexpression of miRNA21 is observed in NAFLD, as well as with radiation exposure [33,61]. A high-calorie diet also induces miRNA21. Alteration of miRNAs might be a clue to understanding how radiation effects are modulated by other factors.

In contrast, calorie restriction to avoid obesity may be useful to protect against radiation effects. Indeed diet control in patients receiving radiotherapies has been considered as a method for ameliorating the adverse effects of radiation [62]. Calorie restriction has been indicated to be useful for inhibiting radiation-induced leukemia in the UNSCEAR 2000 report. In animal experiments, a low calorie diet was reported to suppress liver carcinogenesis [9].

### 3.2. Alcoholic Beverages and Radiation Effects

Drinking alcohol is considered to be related to many diseases, including cancers and excess drinking is a prominent social issues. Indeed, a relationship between alcohol and tumor development has been reported [63]. The carcinogenesis in the liver has been considered to be related to alcohol drinking. Alcohol intake seems to influence radiation effects [14]. Alcohol-related cancers appear to have a higher incidence among nuclear workers. The adverse effects of alcohol beverages are considered to be mostly induced by ethanol. Ethanol has been also observed to influence radiation damage in HepG2 cells [64]. Alcohol is metabolized in the liver and induces oxidative stress [65]. Excess alcohol drinking is known to induce adverse effects on health and can lead to cancer development and liver diseases [66,67]. Excess alcohol intake induces miRNA34 expression [68]. During alcoholic liver diseases, miRNA34 is likely to be a critical mediator in ethanol-activated survival signaling. This miRNA is also an important player in the management of radiotherapy, because it seems to be related to radio-resistance in tumors and radiotoxicity, such as fibrosis in normal tissues [31] (Table 2).

On the other hand, alcoholic beverages are known to have beneficial effects. For example, nutrients in wine such as resveratrol [72], are popular and moderate drinking of wine appears to ameliorate skin damage after radiotherapy [73]. In addition, Japanese sake is gaining popularity around the world and drinking of sake has been reported to rescue mice exposed to high-dose radiation [74]. Ethanol-water appears to rescue mice to some extent but not as effectively as sake. We have also observed beneficial effects from Japanese sake [75]. Mice administered Japanese sake for a month and then irradiated showed greater anti-oxidative abilities in the liver than other mice (control mice, mice administrated sake alone or mice irradiated alone). This alteration has not been observed in the case of mice administrated ethanol-water instead of Japanese sake, so components in sake other than ethanol appear to influence the mouse liver after irradiation. Indeed, some components included in alcohol beverages appear to have beneficial effects [74,76]. In particular, beer, wine and sake reportedly have anti-mutagenic factors [76].

### 3.3. Food Factors and Radio-Sensitivity

As described above, a high calorie diet or alcoholic beverages influence radiation effects on the liver. This modulation may also be due to food factors, including high-calorie food or alcohol beverages. Some of these effects might be beneficial. Some food factors have been suggested to suppress radiation effects in the liver. Radiation protective agents have been developed so far. Amifostine, a radiation-protective agent, is the only drug approved by the FDA in the United States, although it has side effects [77]. In the liver, amifostine is likely to ameliorate radiation-induced damages by suppressing cell death via mechanisms such as autophagy [78]. If beneficial factors are found out in foods, they are expected to be used as radiation-protective agents, as they are considered safe and easy to take. Some factors that influence liver function following radiation are introduced below and provide clues to understanding the effects of radiation on the liver (Table 3).

#### 3.3.1. Resveratrol

Resveratrol is a phytoalexin produced by a variety of plants such as grapes or peanuts and is a known food factor present at high levels in red wine [72]. This substance is considered one of reasons for “French paradox,” the observed inverse relationship between consumption of red wine and incidence of cardiovascular diseases. In terms of effects on the liver following radiation, treatment with resveratrol attenuates decreases in glutathione (GSH) levels and increases in malondialdehyde levels or collagen contents after irradiation [81]. In addition, as an increase in plasma tumor necrosis factor-α (TNFα) concentrations is also suppressed, resveratrol seems to inhibit inflammatory responses after irradiation.

#### 3.3.2. Diallyl Disulfide (DADS)

Garlic is known to have beneficial function [85]. In particular, DADS is a major component of the sulfur-containing compounds in garlic oil and considered to exert anti-cancer effects [86]. The functions of components of garlic oil have been evaluated in terms of radiation protection in the liver [79]. Although some differences existed among indicators of biological effects, radio-protective functions were identified. DADS restored decreased GSH and increases in lipid peroxidation and alkaline phosphatase [79]. DADS may induce 3-mercapopyruvate sulfurtransferase, leading to GSH production [17]. Conversely, DADS has also been reported to have no effect on radiation effects in the liver [87] and further evaluation will be needed in the future.

#### 3.3.3. Curcumin

Curcumin is a main component in turmeric (Curcuma Longa rhizomes), which is used as a spice [88]. Curcumin has various functions [43] and may augment radio-sensitivity [89]. Curcumin lowers levels of reactive oxygen species (ROS) and suppresses activation of nuclear factor-κB (NFκB). In the liver, the constitutive activities of NFκB in cancer cells have been demonstrated to be higher than those in normal cells [90]. Curcumin not only reduces constitutive NFκB activity but also markedly reduces radiation-induced NFκB activities. This may lead to the promotion of radio-sensitivities in liver cancer cells. Indeed, curcumin induces apoptosis in liver cancer cells in combination with radiation [80].

#### 3.3.4. Rutin and Derivative

Rutin is found in onions, apples and red wine and is a well-known flavonoid glycoside [91]. Oral administration of rutin induces anti-oxidative enzymes and protects liver functions and tissue architecture after irradiation [82,83]. Troxerutin, a derivative of rutin [92], may selectively protect normal tissues, including the liver, after irradiation by decreasing lipid peroxidation in normal tissues but not in tumors [93]. Indeed, troxerutin rescues mice after exposure to high-dose irradiation [94].

#### 3.3.5. Ferulic Acid

Ferulic acid (FA) was first isolated from Ferula foetida and is found in foods such as rice, wheat, beans, nuts [95]. Although radiation induces decreased activities of superoxide dismutase and catalase, FA restores these activities [41,84]. FA prevents the production of radiation-induced inflammatory mediators and inflammatory responses, including NFκB signaling. In addition, FA reduces radiation-induced lipid peroxidation. Interestingly, FA also influences DNA damage-repair enzymes [96].

Other food factors such as β-carotene and soy constituents may also be effective against damages or carcinogenesis in the liver after irradiation [12,97,98].

## 4. Perspectives from the View of Radiation Effects on the Liver

Alterations to inflammation-related pathways are definitely important, leading to various liver diseases after irradiation. Inflammation-related pathways including NFκB seem to be between steatosis and liver carcinogenesis. In addition, inflammation is likely to also be related to radiation effects in the liver, modulated by alterations to fatty metabolism induced by obesity or alcohol intake. For example, NFκB may be a target for medical treatment. However, NFκB functions in the liver may be distinct from those in other organs [99]. Modulation of NFκB pathways in the liver should be dealt with very carefully, although suppression of NFκB appears effective against hepatocellular carcinoma cells in combination with radiation [80]. On the other hand, food factors have also been investigated in terms of radiation protection. Antioxidants in foods seem to reduce oxidative stress in our bodies [98,100] and many can also attenuate inflammatory responses.

Effects of irradiation on the liver have been reviewed with consideration of the impact of other factors. Avoiding high-calorie foods and thus suppressing ROS production seems important for health. Using food factors to modulate appropriate pathways associated with radiation effects in the medical field and activities in space may be one method for reducing radiation risks in radiotherapy patients and astronauts.

**Figure 2:**
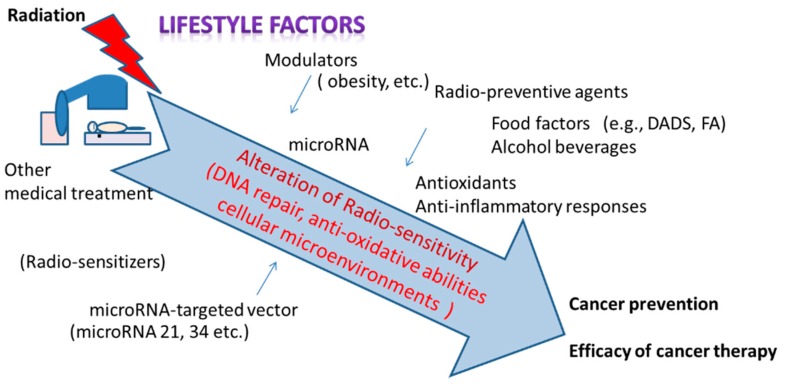
Radio-sensitivity and related factors in radiation protection and cancer radiotherapy.

The liver has numerous functions for maintaining homeostasis. As liver diseases, fatty liver and liver cancer are induced after irradiation. On the other hand, cancer radiotherapies for liver cancers have been increasing recently, although liver tumors are often assumed to be relatively radio-resistant because of the low tolerance of the whole-liver irradiation [101]. The efficacy of therapies and reductions in secondary cancer risk with liver cancer would be helped by improvements including the application of miRNA targeting [102,103]. Particularly miRNA21 and miRNA34a could be promising targets in radiation therapy. Indeed, increased expression of miRNA21 and miRNA34a were reported in human hepatocellular carcinomas and these miRNAs were suggested to be related to cancer recurrence [69,70,71] (Table 2). Understanding the molecular basis of radiation effects in the liver would lead to various applications that will facilitate medical treatment (Figure 2). In modern society, radiation is seeing use in medical fields more and more often. Appropriate lifestyles promote anti-inflammatory or anti-oxidative abilities in the liver, possibly reducing not only metabolic diseases but also the adverse effects of exposure to radiation.

## Figures and Tables

**Table 1:** Alterations in representative molecule expression or activities after irradiation (IR).

Radiation-Induced Responses	Molecules	Radiation Dose (Observed Time After IR)	Fold (Altered Expression or Activities after IR)	Ref (Ref. No.)
Anti-oxidative responses	SOD	4.5Gy (2 h)	2.0 (in activity)	Koiram, et al., 2007 [43]
	catalase	4.5Gy (4 h)	2.0 (in activity)	
Apoptosis-related responses	BclxL	4Gy (3 months)	0.3 (in protein expression)	Nakajima, et al., 2017 [36]
	Caspase3	8Gy (36 h)	1.6 (in activity)	Ozyurt, et al., 2014 [37]
Inflammation-related responses	TNFα	10Gy (24 h)	2.0 (in protein expression)	Das, et al., 2017 [41]
	IL6	10Gy (24 h)	1.8 (in protein expression)	

**Table 2:** Levels and functions of miRNAs in human hepatocellular carcinomas.

miRNAs	Levels in HCC	Characteristics of HCC	Ref. of Data in miRNA Expression (Ref. No.)	Remarkable Functions in HCC (Ref. No.)
miRNA21	High (9.43 ± 0.15) compared to normal liver tissues	-	Meng, et al., 2007 [69]	A cancer-promoting factor [32]
	Higher expression	Poor prognosis	Huang, et al., 2015 [70]	
miRNA34a	High (4.66 ± 0.39) compared to normal liver tissues	-	Meng, et al., 2007 [69]	Controlling of radio-sensitivity [31]
	Lower expression	Early recurrence	Cui, et al., 2015 [71]	

**Table 3:** Modulation of radiation effects in the liver and liver cells by food factors.

Food Factors	Modulation of Radiation Effects in Liver	Ref. (Ref. No.)
DADS	Anti-oxidative stress responses (GSH↑)	Chittezhath and Kuttan 2006 [79]
Curcumin	Anti-oxidative stress responses (SOD↑ etc.)	Koiram, et al., 2007 [43] Hsu, et al., 2015 [80]
	Inflammatory responses (NFκB↓)	
	Apoptosis-related responses (Bcl2↓ etc.)	
Resveratrol	Anti-oxidative stress responses (GSH↑)	Velioğlu-Oğünç, et al., 2009 [81]
	Inflammatory responses (TNFα↓ etc.)	
Rutin	Anti-oxidative stress responses (GSH↑ etc.)	Patil, et al., 2012 [82] Mansour, et al., 2017 [83]
	Inflammatory responses (TNFα↓ etc.)	
FA	Inflammatory responses (TNFα↓ etc.)	Das, et al., 2014 [41] Salem, et al., 2016 [84]
	Anti-oxidative stress responses (SOD↑ etc.)	

Arrows of ↑ or ↓ indicate promotion or suppression by food factors, respectively.

## References

[1] Chiba S., Saito A., Ogawa S., Takeuchi K., Kumano K., Seo S., Suzuki T., Tanaka Y., Saito T., Izutsu K. (2002). Transplantation for accidental acute high-dose total body neutron- and gamma-radiation exposure. Bone Marrow Transpl..

[2] Hawkins M., Dawson L. (2006). Radiation therapy for hepatocellular carcinoma: From palliation to cure. Cancer.

[3] Kim J., Jung Y. (2017). Radiation-induced liver disease: Current understanding and future perspectives. Exp. Mol. Med..

[4] Gonzalez A.B.D., Gilbert E., Curtis R., Inskip P., Kleinerman R., Morton L., Rajaraman P., Little M. (2013). Second solid cancers after radiation therapy: A systematic review of the epidemiologic studies of the radiation dose-response relationship. Int. J. Radiat. Oncol. Biol. Phys..

[5] Krasin M., Constine L., Friedman D., Marks L. (2010). Radiation-related treatment effects across the age spectrum: Differences and similarities or what the old and young can learn from each other. Semin. Radiat. Oncol..

[6] Preston D., Ron E., Tokuoka S., Funamoto S., Nishi N., Soda M., Mabuchi K., Kodama K. (2007). Solid cancer incidence in atomic bomb survivors: 1958–1998. Radiat. Res..

[7] Akahoshi M., Amasaki Y., Soda M., Hida A., Imaizumi M., Nakashima E., Maeda R., Seto S., Yano K. (2003). Effects of radiation on fatty liver and metabolic coronary risk factors among atomic bomb survivors in Nagasaki. Hypertens. Res..

[8] Kennedy A., Davis J., Carlton W., Ware J. (2008). Effects of dietary antioxidant supplementation on the development of malignant lymphoma and other neoplastic lesions in mice exposed to proton or iron-ion radiation. Radiat. Res..

[9] Shang Y., Kakinuma S., Yamauchi K., Morioka T., Kokubo T., Tani S., Takabatake T., Kataoka Y., Shimada Y. (2014). Cancer prevention by adult-onset calorie restriction after infant exposure to ionizing radiation in B6C3F1 male mice. Int. J. Cancer.

[10] Tanaka I.B., Tanaka S., Ichinohe K., Matsushita S., Matsumoto T., Otsu H., Oghiso Y., Sato F. (2007). Cause of death and neoplasia in mice continuously exposed to very low dose rates of gamma rays. Radiat. Res..

[11] Sasaki S. (1991). Influence of the age of mice at exposure to radiation on life-shortening and carcinogenesis. J. Radiat. Res..

[12] Ito A., Watanabe H., Basaran N. (1993). Effects of soy products in reducing risk of spontaneous and neutron-induced liver tumors in mice. Int. J. Oncol..

[13] Khan N., Afaq F., Mukhtar H. (2010). Lifestyle as risk factor for cancer: Evidence from human studies. Cancer Lett..

[14] Akiba S., Mizuno S. (2012). The third analysis of cancer mortality among Japanese nuclear workers, 1991-2002: Estimation of excess relative risk per radiation dose. J. Radiol. Prot..

[15] Jonscher K., Alfonso-Garcia A., Suhalim J., Orlicky D., Potma E., Ferguson V., Bouxsein M., Bateman T., Stodieck L., Levi M. (2016). Spaceflight Activates Lipotoxic Pathways in Mouse Liver. PLoS ONE.

[16] Kojima S., Matsuki O., Nomura T., Kubodera A., Honda Y., Honda S., Tanooka H., Wakasugi H., Yamaoka K. (1998). Induction of mRNAs for glutathione synthesis-related proteins in mouse liver by low doses of gamma-rays. Biochim. Biophys. Acta.

[17] Nakajima T. (2015). Roles of Sulfur Metabolism and Rhodanese in Detoxification and Anti-Oxidative Stress Functions in the Liver: Responses to Radiation Exposure. Med. Sci. Monit..

[18] Uehara Y., Ikehata H., Komura J., Ito A., Ogata M., Itoh T., Hirayama R., Furusawa Y., Ando K., Paunesku T. (2008). Absence of *Ku70* gene obliterates X-ray-induced lacZ mutagenesis of small deletions in mouse tissues. Radiat. Res..

[19] Jirtle R., Michalopoulos G., Strom S., DeLuca P., Gould M. (1984). The survival of parenchymal hepatocytes irradiated with low and high LET radiation. Br. J. Cancer.

[20] Moriconi F., Christiansen H., Raddatz D., Dudas J., Hermann R., Rave-Fränk M., Sheikh N., Saile B., Hess C., Ramadori G. (2008). Effect of radiation on gene expression of rat liver chemokines: In vivo and in vitro studies. Radiat. Res..

[21] Amanzada A., Malik I., Nischwitz M., Sultan S., Naz N., Ramadori G. (2011). Myeloperoxidase and elastase are only expressed by neutrophils in normal and in inflamed liver. Histochem. Cell Biol..

[22] Malik I., Moriconi F., Sheikh N., Naz N., Khan S., Dudas J., Mansuroglu T., Hess C., Rave-Fränk M., Christiansen H. (2010). Single-dose gamma-irradiation induces up-regulation of chemokine gene expression and recruitment of granulocytes into the portal area but not into other regions of rat hepatic tissue. Am. J. Pathol..

[23] Shang Y., Sawa Y., Blyth B., Tsuruoka C., Nogawa H., Shimada Y., Kakinuma S. (2017). Radiation Exposure Enhances Hepatocyte Proliferation in Neonatal Mice but not in Adult Mice. Radiat. Res..

[24] Yu X., Lu L., Wen S., Wang Y. (2009). The effects of Fhit on tumorigenesis after multi-exposure to low-dose radiation. Int. J. Clin. Exp. Med..

[25] Semba S., Huebner K. (2006). Protein expression profiling identifies cyclophilin A as a molecular target in Fhit-mediated tumor suppression. Mol. Cancer Res..

[26] Pawlik A., Delmar P., Bosse S., Sainz L., Petat C., Pietu G., Thierry D., Roux D.T.-L. (2009). Changes in transcriptome after in vivo exposure to ionising radiation reveal a highly specialised liver response. Int. J. Radiat. Biol..

[27] Uehara Y., Ito Y., Taki K., Nenoi M., Ichinohe K., Nakamura S., Tanaka S., Oghiso Y., Tanaka K., Matsumoto T. (2010). Gene expression profiles in mouse liver after long-term low-dose-rate irradiation with gamma rays. Radiat. Res..

[28] Roudkenar M., Li L., Baba T., Kuwahara Y., Nakagawa H., Wang L., Kasaoka S., Ohkubo Y., Ono K., Fukumoto M. (2008). Gene expression profiles in mouse liver cells after exposure to different types of radiation. J. Radiat. Res..

[29] Miousse I., Kutanzi K., Koturbash I. (2017). Effects of ionizing radiation on DNA methylation: From experimental biology to clinical applications. Int. J. Radiat. Biol..

[30] Kovalchuk O., Burke P., Besplug J., Slovack M., Filkowski J., Pogribny I. (2004). Methylation changes in muscle and liver tissues of male and female mice exposed to acute and chronic low-dose X-ray-irradiation. Mutat. Res. Fund. Mol. Mech. Mutagen..

[31] Lacombe J., Zenhausern F. (2017). Emergence of miR-34a in radiation therapy. Crit. Rev. Oncol. Hematol..

[32] Shu X., Fan C., Long B., Zhou X., Wang Y. (2016). The anti-cancer effects of cisplatin on hepatic cancer are associated with modulation of miRNA-21 and miRNA-122 expression. Eur. Rev. Med. Pharmacol. Sci..

[33] Zhu Y., Yu X., Fu H., Wang H., Wang P., Zheng X., Wang Y. (2010). MicroRNA-21 is involved in ionizing radiation-promoted liver carcinogenesis. Int. J. Clin. Exp. Med..

[34] Lu J., Chen C., Hao L., Zheng Z., Zhang N., Wang Z. (2016). MiRNA expression profile of ionizing radiation-induced liver injury in mouse using deep sequencing. Cell Biol. Int..

[35] Bakshi M., Azimzadeh O., Barjaktarovic Z., Kempf S., Merl-Pham J., Hauck S., Buratovic S., Eriksson P., Atkinson M., Tapio S. (2015). Total body exposure to low-dose ionizing radiation induces long-term alterations to the liver proteome of neonatally exposed mice. J. Proteome Res..

[36] Nakajima T., Wang B., Ono T., Uehara Y., Nakamura S., Ichinohe K., Braga-Tanaka I., Tanaka S., Tanaka K., Nenoi M. (2017). Differences in sustained alterations in protein expression between livers of mice exposed to high-dose-rate and low-dose-rate radiation. J. Radiat. Res..

[37] Özyurt H., Özden A., Çevik Ö., Özgen Z., Cadirci S., Elmas M., Ercan F., Şener G., Gören M. (2014). Investigation into the role of the cholinergic system in radiation-induced damage in the rat liver and ileum. J. Radiat. Res..

[38] Teoh N., Pyakurel P., Dan Y., Swisshelm K., Hou J., Mitchell C., Fausto N., Gu Y., Farrell G. (2010). Induction of p53 renders ATM-deficient mice refractory to hepatocarcinogenesis. Gastroenterology.

[39] Mihailović M., Milosević V., Grigorov I., Poznanović G., Ivanović-Matić S., Grdović N., Bogojević D. (2009). The radioprotective effect of alpha2-macroglobulin: A morphological study of rat liver. Med. Sci. Monit..

[40] Zhou D., Brown S., Yu T., Chen G., Barve S., Kang B., Thompson J. (1999). A high dose of ionizing radiation induces tissue-specific activation of nuclear factor-kappaB in vivo. Radiat. Res..

[41] Das U., Manna K., Sinha M., Datta S., Das D., Chakraborty A., Ghosh M., Saha K., Dey S. (2014). Role of ferulic acid in the amelioration of ionizing radiation induced inflammation: A murine model. PLoS ONE.

[42] Bogojević D., Poznanović G., Grdović N., Grigorov I., Vidaković M., Dinić S., Mihailović M. (2011). Administration of rat acute-phase protein α2-macroglobulin before total-body irradiation initiates cytoprotective mechanisms in the liver. Radiat. Environ. Biophys..

[43] Koiram P., Veerapur V., Kunwar A., Mishra B., Barik A., Priyadarsini I., Mazhuvancherry U. (2007). Effect of curcumin and curcumin copper complex (1:1) on radiation-induced changes of anti-oxidant enzymes levels in the livers of Swiss albino mice. J. Radiat. Res..

[44] Grant E.J., Ozasa K., Preston D.L., Suyama A., Shimizu Y., Sakata R., Sugiyama H., Pham T.M., Cologne J., Yamada M. (2012). Effects of radiation and lifestyle factors on risks of urothelial carcinoma in the Life Span Study of atomic bomb survivors. Radiat. Res..

[45] Nagano J., Kono S., Preston D., Moriwaki H., Sharp G., Koyama K., Mabuchi K. (2000). Bladder-cancer incidence in relation to vegetable and fruit consumption: A prospective study of atomic-bomb survivors. Int. J. Cancer.

[46] Boaventura P., Durães C., Mendes A., Costa N., Chora I., Ferreira S., Araújo E., Lopes P., Rosa G., Marques P. (2018). Is Low-Dose Radiation Exposure a Risk Factor for Atherosclerotic Disease?. Radiat. Res..

[47] Braga-Tanaka I., Tanaka S., Kohda A., Takai D., Nakamura S., Ono T., Tanaka K., Komura J. (2018). Experimental studies on the biological effects of chronic low dose-rate radiation exposure in mice: Overview of the studies at the Institute for Environmental Sciences. Int. J. Radiat. Biol..

[48] Zoller H., Tilg H. (2016). Nonalcoholic fatty liver disease and hepatocellular carcinoma. Metabolism.

[49] Martius G., Alwahsh S., Rave-Fränk M., Hess C., Christiansen H., Ramadori G., Malik I. (2014). Hepatic fat accumulation and regulation of FAT/CD36: An effect of hepatic irradiation. Int. J. Clin. Exp. Pathol..

[50] Wolin K., Carson K., Colditz G. (2010). Obesity and cancer. Oncogene.

[51] Nakamura A., Terauchi Y. (2013). Lessons from Mouse Models of High-Fat Diet-Induced NAFLD. Int. J. Mol. Sci..

[52] Saito Y., Kuwahara Y., Yamamoto Y., Suzuki M., Fukumoto M., Yamamoto F. (2018). ddY Mice Fed 10% Fat Diet Exhibit High p27KIP Expression and Delayed Hepatocyte DNA Synthesis During Liver Regeneration. Metab. Syndr. Relat. Disord..

[53] Vares G., Wang B., Ishii-Ohba H., Nenoi M., Nakajima T. (2014). Diet-induced obesity modulates epigenetic responses to ionizing radiation in mice. PLoS ONE.

[54] Toprani S., Das B. (2015). Role of base excision repair genes and proteins in gamma-irradiated resting human peripheral blood mononuclear cells. Mutagenesis.

[55] Pang J., Xi C., Dai Y., Gong H., Zhang T. (2012). Altered expression of base excisions repair genes in response to high glucose-induced oxidative stress in HepG2 hepatocytes. Med. Sci. Monit..

[56] Shimizu Y., Kodama K., Nishi N., Kasagi F., Suyama A., Soda M., Grant E.J., Sugiyama H., Sakata R., Moriwaki H. (2010). Radiation exposure and circulatory disease risk: Hiroshima and Nagasaki atomic bomb survivor data, 1950–2003. BMJ.

[57] Marseglia L., Manti S., D’Angelo G., Nicotera A., Parisi E., Di Rosa G., Gitto E., Arrigo T. (2015). Oxidative stress in obesity: A critical component in human diseases. Int. J. Mol. Sci..

[58] Bonomini F., Rodella L.F., Rezzani R. (2015). Metabolic syndrome, aging and involvement of oxidative stress. Aging Dis..

[59] Mercer J.R., Cheng K.K., Figg N., Gorenne I., Mahmoudi M., Griffin J., Vidal-Puig A., Logan A., Murphy M.P., Bennett M. (2010). DNA damage links mitochondrial dysfunction to atherosclerosis and the metabolic syndrome. Circ. Res..

[60] Stewart F.A., Heeneman S., Te Poele J., Kruse J., Russell N.S., Gijbels M., Daemen M. (2006). Ionizing radiation accelerates the development of atherosclerotic lesions in ApoE-/-mice and predisposes to an inflammatory plaque phenotype prone to hemorrhage. Am. J. Pathol..

[61] López-Riera M., Conde I., Quintas G., Pedrola L., Zaragoza Á., Perez-Rojas J., Salcedo M., Benlloch S., Castell J., Jover R. (2018). Non-invasive prediction of NAFLD severity: A comprehensive, independent validation of previously postulated serum microRNA biomarkers. Sci. Rep..

[62] Klement R., Champ C. (2014). Calories, carbohydrates, and cancer therapy with radiation: Exploiting the five R’s through dietary manipulation. Cancer Metastasis Rev..

[63] Praud D., Rota M., Rehm J., Shield K., Zatoński W., Hashibe M., Vecchia C.L., Boffetta P. (2016). Cancer incidence and mortality attributable to alcohol consumption. Int. J. Cancer.

[64] Ogony J., Matthews R., Anni H., Shannon K., Ercal N. (2008). The mechanism of elevated toxicity in HepG2 cells due to combined exposure to ethanol and ionizing radiation. J. Appl. Toxicol..

[65] Das S., Vasudevan D. (2007). Alcohol-induced oxidative stress. Life Sci..

[66] Inoue M., Tsugane S. (2005). Impact of alcohol drinking on total cancer risk: Data from a large-scale population-based cohort study in Japan. Br. J. Cancer.

[67] Orman E.S., Odena G., Bataller R. (2013). Alcoholic liver disease: Pathogenesis, management, and novel targets for therapy. J. Gastroenterol. Hepatol..

[68] McDaniel K., Herrera L., Zhou T., Francis H., Han Y., Levine P., Lin E., Glaser S., Alpini G., Meng F. (2014). The functional role of microRNAs in alcoholic liver injury. J. Cell. Mol. Med..

[69] Meng F., Henson R., Wehbe-Janek H., Ghoshal K., Jacob S., Patel T. (2007). MicroRNA-21 regulates expression of the PTEN tumor suppressor gene in human hepatocellular cancer. Gastroenterology.

[70] Huang C., Yu W., Cui H., Wang Y., Zhang L., Han F., Huang T. (2015). Increased expression of miR-21 predicts poor prognosis in patients with hepatocellular carcinoma. Int. J. Clin. Exp. Pathol..

[71] Cui X., Wu Y., Wang Z., Liu X., Wang S., Qin C. (2015). MicroRNA-34a expression is predictive of recurrence after radiofrequency ablation in early hepatocellular carcinoma. Tumor Biol..

[72] Aggarwal B., Bhardwaj A., Aggarwal R., Seeram N., Shishodia S., Takada Y. (2004). Role of resveratrol in prevention and therapy of cancer: Preclinical and clinical studies. Anticancer Res..

[73] Morganti A.G., Digesu C., Panunzi S., De Gaetano A., Macchia G., Deodato F., Cece M.G., Cirocco M., Di Castelnuovo A., Iacoviello L. (2009). Radioprotective effect of moderate wine consumption in patients with breast carcinoma. Int. J. Radiat. Oncol. Biol. Phys..

[74] Takizawa Y., Yamashita J., Ishigouka S. (2014). Protective Effect of Japanese Sake against Ionizing X-irradiation in Mice. Radioisotopes.

[75] Nakajima T., Vares G., Wang B., Nenoi M. (2016). Chronic Intake of Japanese Sake Mediates Radiation-Induced Metabolic Alterations in Mouse Liver. PLoS ONE.

[76] Arimoto-Kobayashi S., Sugiyama C., Harada N., Takeuchi M., Takemura M., Hayatsu H. (1999). Inhibitory effects of beer and other alcoholic beverages on mutagenesis and DNA adduct formation induced by several carcinogens. J. Agric. Food Chem..

[77] Singh V., Fatanmi O., Wise S., Newman V., Romaine P., Seed T. (2016). The potentiation of the radioprotective efficacy of two medical countermeasures, gamma-tocotrienol and amifostine, by a combination prophylactic modality. Radiat. Prot. Dosim..

[78] Koukourakis M.I., Giatromanolaki A., Fylaktakidou K., Kouroupi M., Sivridis E., Zois C., Kalamida D., Mitrakas A., Pouliliou S., Karagounis I. (2018). Amifostine Protects Mouse Liver Against Radiation-induced Autophagy Blockage. Anticancer Res..

[79] Chittezhath M., Kuttan G. (2006). Radioprotective activity of naturally occurring organosulfur compounds. Tumori.

[80] Hsu F.-T., Liu Y.-C., Liu T.-T., Hwang J.-J. (2015). Curcumin Sensitizes Hepatocellular Carcinoma Cells to Radiation via Suppression of Radiation-Induced NF-κB Activity. BioMed Res. Int..

[81] Velioğlu-Oğünç A., Sehirli O., Toklu H., Ozyurt H., Mayadağli A., Ekşioğlu-Demiralp E., Erzik C., Cetinel S., Yeğen B., Sener G. (2009). Resveratrol protects against irradiation-induced hepatic and ileal damage via its anti-oxidative activity. Free Radic. Res..

[82] Patil S., Somashekarappa H., Rajashekhar K. (2012). Radiomodulatory role of Rutin and Quercetin in Swiss Albino mice exposed to the whole body gamma radiation. Indian J. Nucl. Med..

[83] Mansour S., El-Marakby S., Moawed F. (2017). Ameliorative effects of rutin on hepatic encephalopathy-induced by thioacetamide or gamma irradiation. J. Photochem. Photobiol. B..

[84] Salem A., Mohammaden T., Ali M., Mohamed E., Hasan H. (2016). Ellagic and ferulic acids alleviate gamma radiation and aluminium chloride-induced oxidative damage. Life Sci..

[85] Butt M., Sultan M., Butt M., Iqbal J. (2009). Garlic: Nature’s protection against physiological threats. Crit. Rev. Food Sci. Nutr..

[86] Yi L., Su Q. (2013). Molecular mechanisms for the anti-cancer effects of diallyl disulfide. Food Chem. Toxicol..

[87] Tenkanidiyoor Y., Vasudeva V., Rao S., Gowda D., Rao C., Sanjeev G., Nalilu S. (2016). Haematopoietic, Antioxidant and Membrane Stabilizing Property of Diallyl Disulphide in Irradiated Mice. J. Clin. Diagn. Res..

[88] Maheshwari R., Singh A., Gaddipati J., Srimal R. (2006). Multiple biological activities of curcumin: A short review. Life Sci..

[89] Verma V. (2016). Relationship and interactions of curcumin with radiation therapy. World J. Clin. Oncol..

[90] Li W., Tan D., Zenali M., Brown R. (2010). Constitutive activation of nuclear factor-kappa B (NF-kB) signaling pathway in fibrolamellar hepatocellular carcinoma. Int. J. Clin. Exp. Pathol..

[91] Slámová K., Kapešová J., Valentová K. (2018). “Sweet Flavonoids”: Glycosidase-Catalyzed Modifications. Int. J. Mol. Sci..

[92] Wang Y., Wei S., Chen L., Pei J., Wu H., Pei Y., Chen Y., Wang D. (2017). Transcriptomic analysis of gene expression in mice treated with troxerutin. PLoS ONE.

[93] Maurya D., Salvi V., Nair C.K. (2004). Radioprotection of normal tissues in tumor-bearing mice by troxerutin. J. Radiat. Res..

[94] Ping X., Junqing J., Junfeng J., Enjin J. (2012). Radioprotective effects of troxerutin against gamma irradiation in mice liver. Int. J. Radiat. Biol..

[95] Kumar N., Pruthi V. (2014). Potential applications of ferulic acid from natural sources. Biotechnol. Rep..

[96] Das U., Manna K., Khan A., Sinha M., Biswas S., Sengupta A., Chakraborty A., Dey S. (2017). Ferulic acid (FA) abrogates γ-radiation induced oxidative stress and DNA damage by up-regulating nuclear translocation of Nrf2 and activation of NHEJ pathway. Free Radic. Res..

[97] El-Habit O., Saada H., Azab K., Abdel-Rahman M., El-Malah D. (2000). The modifying effect of beta-carotene on gamma radiation-induced elevation of oxidative reactions and genotoxicity in male rats. Mutat. Res..

[98] Kennedy A. (2009). Factors that modify radiation-induced carcinogenesis. Health Phys..

[99] Muriel P. (2009). NF-kappaB in liver diseases: A target for drug therapy. J. Appl. Toxicol..

[100] Kennedy A.R., Ware J.H., Carlton W., Davis J.G. (2011). Suppression of the later stages of radiation-induced carcinogenesis by antioxidant dietary formulations. Radiat. Res..

[101] Rim C., Yoon W. (2018). Leaflet manual of external beam radiation therapy for hepatocellular carcinoma: A review of the indications, evidences, and clinical trials. Onco Targets Ther..

[102] Dutta R., Mahato R. (2017). Recent advances in hepatocellular carcinoma therapy. Pharmacol. Ther..

[103] Erstad D., Fuchs B., Tanabe K. (2018). Molecular signatures in hepatocellular carcinoma: A step toward rationally designed cancer therapy. Cancer.

